# Comparing built-up area datasets to assess urban exposure to coastal hazards in Europe

**DOI:** 10.1038/s41597-024-03339-4

**Published:** 2024-05-15

**Authors:** Hedda Bonatz, Lena Reimann, Athanasios T. Vafeidis

**Affiliations:** 1 Coastal Risks and Sea-level Rise Research Group, Department of Geography, Christian-Albrechts University Kiel, Ludewig-Meyn-Straße 8, 24118 Kiel, Germany; 2 Institute for Environmental Studies, VU University, De Boelelaan 1087, 1081 HV Amsterdam, The Netherlands

**Keywords:** Environmental impact, Environmental impact, Natural hazards

## Abstract

Information on urban land use, beyond the urban-rural dichotomy, can improve the assessment of potential impacts of coastal hazards by refining estimates of damages and supporting adaptation planning. However, the lack of a consistent definition of “urban” in previous studies has led to exposure estimates that vary considerably. Here, we explore the sensitivity of exposed population and built-up area in four settlement types, defined by four different built-up area datasets. We find large differences in the exposed population of up to 65% (127 million people) in the “Urban” class. The exposure estimates are highly sensitive to the density thresholds used to distinguish the settlement types, with a difference in exposed urban population of up to 53.5 million people when the threshold varies by 10%. We attribute the high sensitivity of the exposure estimates to the varying definitions of built-up area of the underlying datasets. We argue that the definition of urban land is crucial for coastal impact assessments and make recommendations for the use of the analyzed datasets.

## Introduction

Europe’s coast is highly urbanized, with 40% of the coastal population living in urban areas^1^. Especially in Western Europe the population density at the coast is much higher (328 people/km²) compared to the global average (241 people/km^2^)^1^. High population densities, agglomerations of large urban settlements, critical infrastructure and the concentration of vulnerable assets and communities render urban coastal areas highly exposed to climatic hazards^1–9^. These hazards are projected to increase in intensity and frequency through anthropogenic climate change^10–12^. Exposure and risk to climatic hazards are particularly exacerbated due to fast socio-economic development, high urbanization levels and coastal migration^2,4,13–17^.

The impacts of climate hazards are assessed and managed differently in urban as compared to rural areas. Urban areas contain large parts of the global economy^18^ and are characterized by more complex and developed infrastructures, which makes them more susceptible to hazards compared to rural areas^3,5–7^. In fact, 90% of the estimated damages at the European coast are estimated to emerge from urban areas^19,20^. Furthermore, the implementation of suitable adaptation techniques is influenced by the structures of human settlements^19,21^. For instance, nature-based adaptation is likely to be implemented in sparsely populated locations with sufficient accommodation space^22^, whereas hard protection infrastructure is still considered the most cost efficient in highly urbanized areas^5,23–25^; while for other locations socio-economic conditions are relevant to discuss options such as setback zones or retreat^20,26–29^.

Most exposure assessments distinguish between urban and rural settlement types to evaluate differences in exposure to climate hazards^1,2,30^. However, large parts of the inhabited land do not fit the classical urban versus rural definitions as they cannot be clearly defined by the characteristics that distinguish urban from rural areas, such as population size or social structure^31–33^. Thus, a distinction of additional settlement types can refine the evaluation and management of damages, and therefore facilitate the development of customized adaptation paths^33–36^, thus leading to improved estimates of future projections of population and socio-economic development^17,37–39^. In the context of the UN Sustainable Development Goals (SDGs) it is recommended to consistently extend the dichotomous urban-rural classification to guarantee international comparability^32,40^.

A notable challenge in previous assessments has been the substantial variation in exposure estimates due to differing underlying data on population and land area^21,30,41–43^. These differences likely stem from different data characteristics, such as the spatial resolution or the type of satellite imagery employed for producing the datasets^30,44–46^. In urban analyses the main issue involves the often unclear definition of ‘urban’ or ‘built-up’, due to the lack of a consistent definition in the literature^7,47^. This complicates the comparison of the results of urban analyses^40,43,48^ and raises the need for a harmonized approach to define distinct settlement types^40^. An attempt to address this limitation is the ‘Degree of Urbanization (DEGURBA)’ method, which uniformly distinguishes several different urban classes based on the population density and agglomeration size^40^.

In our study we systematically explore and quantify the differences in exposure distribution of built-up land and population in different settlement types in coastal Europe using different data defining the settlement types. As we aim for a definition of settlement types independent from population numbers we distinguish settlement types based on built-up area densities and not according to the DEGURBA. We define four different settlement types based on density of built-up area, using four different built-up area proxy datasets commonly employed in research (Global Human Settlement – Built-up Surface grid (GHS-BUILT-S R2022A), the European Settlement Map (ESM), the World Settlement Footprint (WSF) and the Global Man-made Impervious Surface (GMIS)). We use a single population dataset, namely WorldPop unconstrained, to assess exposed population, to specifically focus on the impact of different built-up area datasets on exposure estimates. Following the study of Mac Manus *et al*.^43^, we apply their proposed density thresholds to differentiate the different settlement types: “Urban”, “Suburban”, “Rural Built-up”, “Rural No Built-up”. We evaluate our estimates by comparing the results with regional built-up area data in six European cities and assess the sensitivity of the data to changes in the density thresholds of the built-up area classes. A detailed methodological description can be found in the Methods section. Based on our results, we provide recommendations for the use of built-up area data to delineate settlement types in risk assessments; and emphasize the importance of considering built-up area definitions in the data selection process and the need to overcome the urban-rural dichotomy by including additional settlement types.

## Results

### Exposure in different settlement types across Europe

**Fig. 1:**
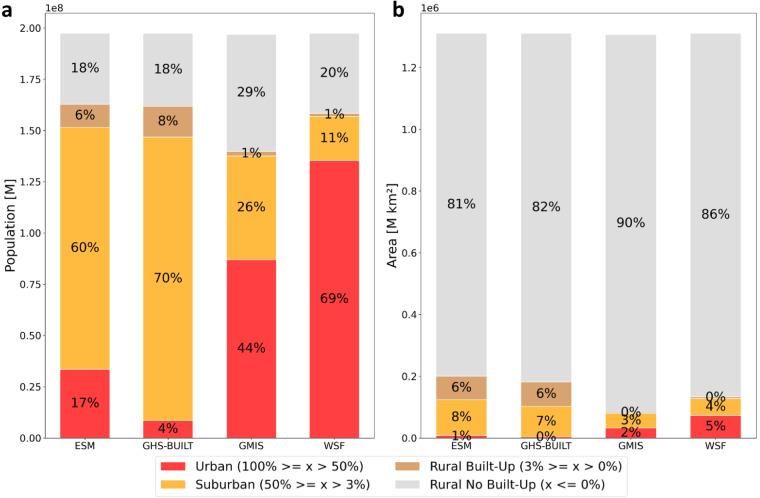
Absolute and relative exposed population(**a**) and area (**b**) per settlement type and built-up proxy dataset.

Figure 1 shows differences in the distribution of coastal population (a) and land area (b) in different settlement types depending on the built-up area dataset used. We find the largest differences in the population shares (Fig. 1a) of the “Urban” and “Suburban” classes across datasets. WSF and GMIS distribute 69% and 44% of the 197 million people living at the European coast in the “Urban” class, respectively. Accordingly, the population shares of the “Suburban” class are 11% for the WSF data and 26% for the GMIS. Reversely, ESM and GHS-BUILT have the highest population share in the “Suburban” class (ESM: 60%, GHS-BUILT: 70%) and lower shares in the “Urban” class (ESM: 17%, GHS-BUILT: 4%). This means the exposed population in the “Urban” class varies by up to 65% between the WSF and GHS-BUILT data and for the “Suburban” class the population share for GHS-BUILT is six times higher than for the WSF data. In absolute terms, the exposed population differs by up to 127 million people in the “Urban” class and 117 million people in the “Suburban” class between the datasets. The shares of the population in “Rural Built-up” areas are relatively small for all datasets, however WFS and GMIS have the smallest rural share with around 1% opposed to 6 to 8% for the ESM and GHS-BUILT data. Most datasets indicate a similarly high share of population living in “Rural No-Built-up” areas of approximately 20% (only exception is GMIS with 30%). Disregarding the different settlement types, we observe that all analyzed datasets distribute a similar population share of around 80% in built-up areas. The built-up area estimates (Fig. 1b) are similarly distributed across datasets and settlement types as the population. GMIS (2.5%) and WSF (5.6%) show higher area share in the “Urban”, and GHS-BUILT (7.5%) and ESM (8.8%) in “Suburban” areas, even though the differences are not as distinct between the settlement types as about 80% to 90% of the area is classified as “Rural No Built-up”.

As expected, we find the highest population densities in the “Urban” class (between 1800 to 3900 people/km²), followed by the “Suburban” (390–1400 people/km²) and the “Rural Built-up” class (140–670 people/km²) for all datasets. However, the population densities vary up to five times (i.e. “Rural Built-up class”) depending on the dataset. The population densities for the settlement types at the coast are up to 500 people per km² higher (“Urban” class) than for the entire Europe, including coastal and inland locations, affirming higher population densities in coastal locations.

### Regional evaluation of the built-up proxy datasets

**Fig. 2:**
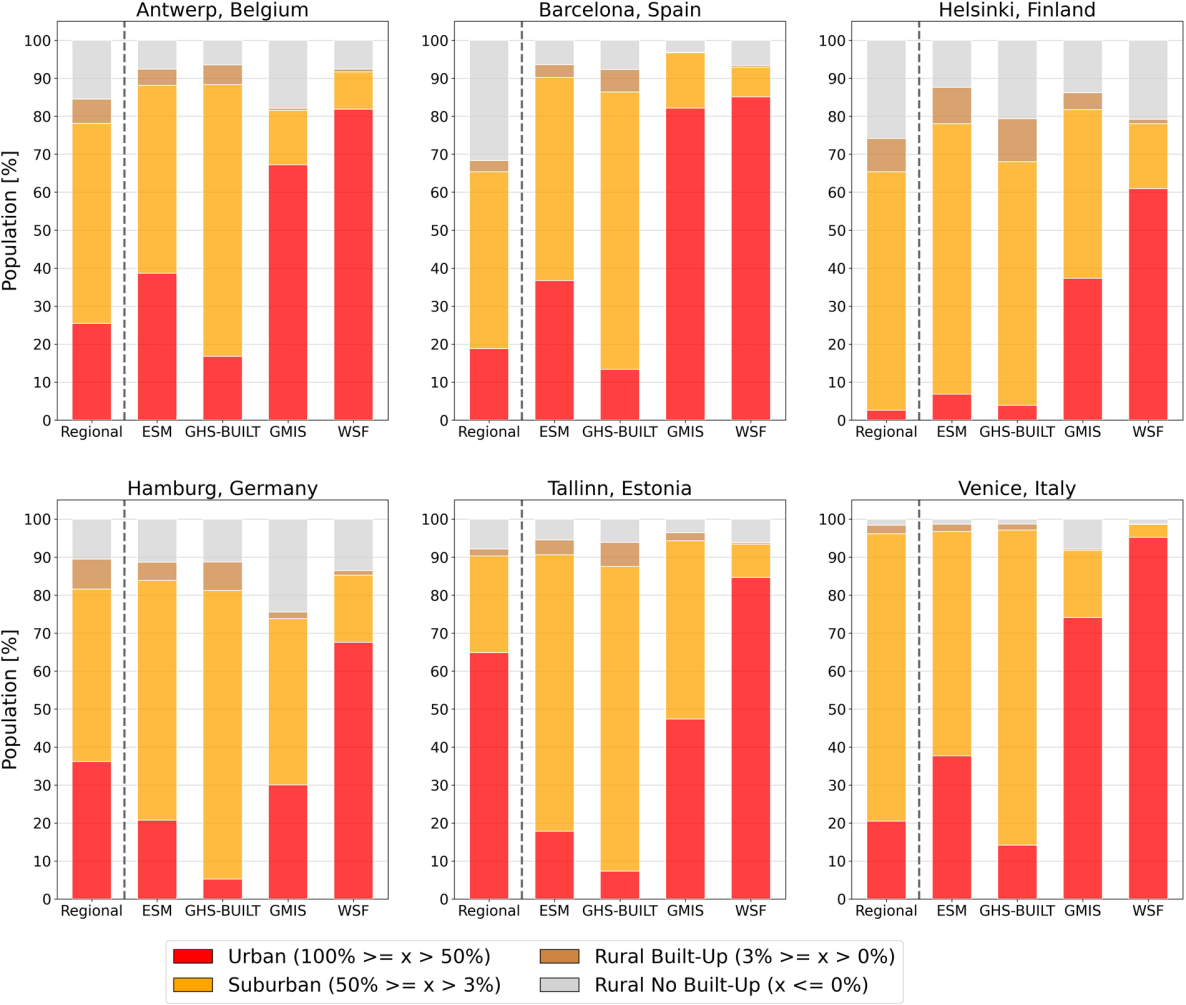
Comparison of the relative population shares per settlement type estimated by the built-up proxy data and the regional data for six European cities.

Figure 2 shows the distribution of exposed population within the settlement types for the regional built-up evaluation data (left bar per panel) as opposed to the built-up proxy estimates for the six study sites in Europe (i.e. Antwerp, Barcelona, Helsinki, Hamburg, Tallinn, Venice). Similar to the Europe-wide estimates, WSF and GMIS distribute significantly higher population shares in the “Urban” class across the six cities, whereas for GHS-BUILT and ESM most people are located in “Suburban” areas.

While no exposure estimates of the built-up proxy data entirely reflect the population distribution of the regional data, we see two different tendencies when comparing the regional data with the built-up proxy data: (i) for Antwerp, Barcelona, Helsinki and Venice the population distribution within each built-up area class matches the regional data for GHS-BUILT and ESM better, (ii) for Tallinn and Hamburg the population distribution best matches the regional data for WSF and GMIS.

In detail, in Antwerp the population share in the “Urban” class is between 8% lower (GHS-BUILT) and 67% higher (WSF) than the regional data and between 43% lower (WSF) and 21% higher (GHS-BUILT) for the “Suburban” class. We observe similar differences between the built-up proxy data and the regional data in Barcelona (“Urban”: GHS-BUILT 6% lower; WSF 66% higher, “Suburban”: WSF 39% lower; GHS-BUILT 26% higher), Venice (“Urban”: GHS-BUILT 6% lower; WSF 75% higher, “Suburban”: WSF 73% lower; GHS-BUILT 7% higher)and Helsinki (“Urban”: GHS-BUILT 1% higher; WSF 58% higher, “Suburban”: WSF 45% lower; ESM: 8% higher). In Tallinn and Hamburg, we see the contrary tendency. In Tallinn the population share in the “Urban” class for GHS-BUILT is 58% lower than the regional data and 20% higher for WSF, and in the “Suburban” class 16% lower for the WSF and 55% higher for the GHS-BUILT. In Hamburg the population share deviates between −31% (GHS-BUILT) and +32% (WSF) from the regional data in the “Urban” class and −27% (WSF) and +31% (GHS-BUILT) in the “Suburban” class. Here the ESM data matches the best with the regional data, with 15% population difference in the “Urban” class and 18% in the “Suburban” class. The differences in the two rural classes are smaller and do not change the overall tendencies. In terms of built-up area shares per settlement type we observe larger differences in the total detected built-up area between the datasets, assuming that these differences also contribute to the varying shares of exposed population (Supplementary Figure 1).

**Fig. 3:**
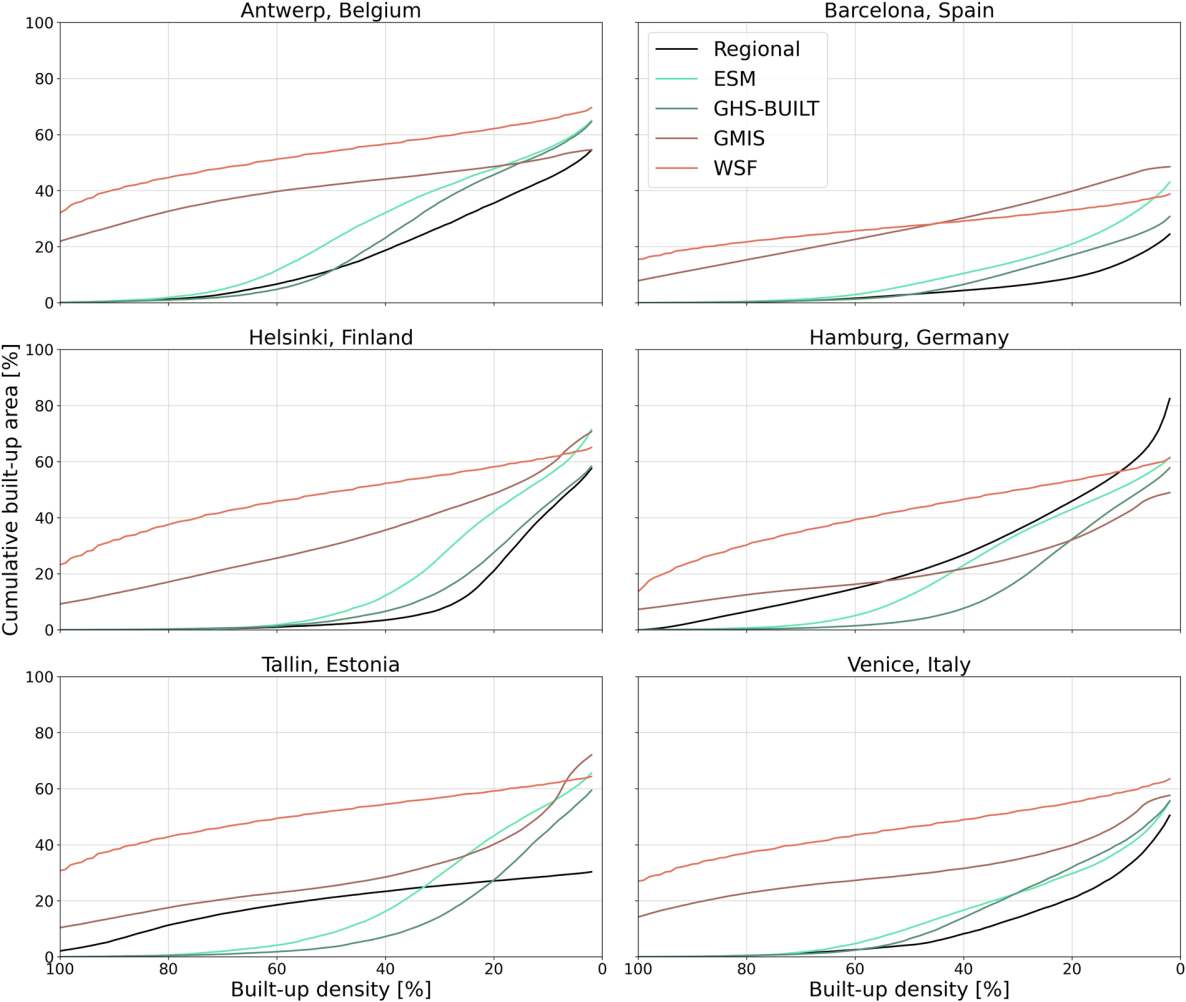
Cumulative built-up area density curves indicating the built-up area share per built-up density percentage point. The black line in the plots represents the trend of the regional data.

To identify the built-up densities that are in best accordance with the regional data and evaluate the reliability of the class thresholds, we analyze the area share per built-up area density percentage point with a cumulative built-up density curve. Figure 3 shows the cumulative built-up area share for the regional and built-up proxy data in relation to the built-up density. We observe the same tendencies discussed earlier: (i) the trends for GHS-BUILT and ESM show a similar pattern as those based on the regional data in Antwerp, Barcelona, Helsinki and Venice; (ii) for Tallinn and Hamburg the WSF and GMIS data appear to fit best with the regional data.

However, we also observe that the accordance between the regional data and the built-up proxy data varies along the range of built-up densities. Generally, we see a better accordance for higher built-up densities from 100% to around 60%. This is particularly visible in Antwerp, Barcelona, Venice and Helsinki, where the cumulative built-up area for GHS-BUILT and ESM only exceed the regional data for built-up densities below 60%. In Tallinn and Hamburg this observation is less clear, as the cumulative built-up area shares for WSF and GMIS are more homogenously distributed across the built-up densities, compared to the regional data. In both cities we see a better accordance of the GMIS data with the regional data especially for built-up densities above 50%, but the cumulative built-up area is overestimating the distribution of the regional data by up to 10%. The WSF data follow a similar distribution as the regional data for higher built-up densities, but overestimate the regional data by around 30%. For lower densities the distribution of the built-up area in Tallinn and Hamburg do not follow a visible trend that can be related to the regional data.

Overall, the comparison of the exposure estimates with the regional built-up area data shows that it is difficult to identify a built-up proxy dataset that is in complete accordance with the regional data. Nevertheless, we observe similarities for specific settlement types and densities. For example, particularly for built-up area densities above 60%, the estimates of exposed built-up area by GHS-BUILT and ESM fit better to the regional data in Antwerp, Barcelona, Venice and Helsinki; whereas WSF and GMIS show better accordance for Tallinn and Hamburg. It should be noted that the described results are biased by the initial choice of regional data because the regional data are not harmonized across the study sites. Consequently, they more accurately represent the distribution of built-up proxy data with similar characteristics. Further details on this matter can be found in the discussion section.

### Sensitivity of the population share to changes in the density thresholds differs by up to 22% between the datasets

**Fig. 4:**
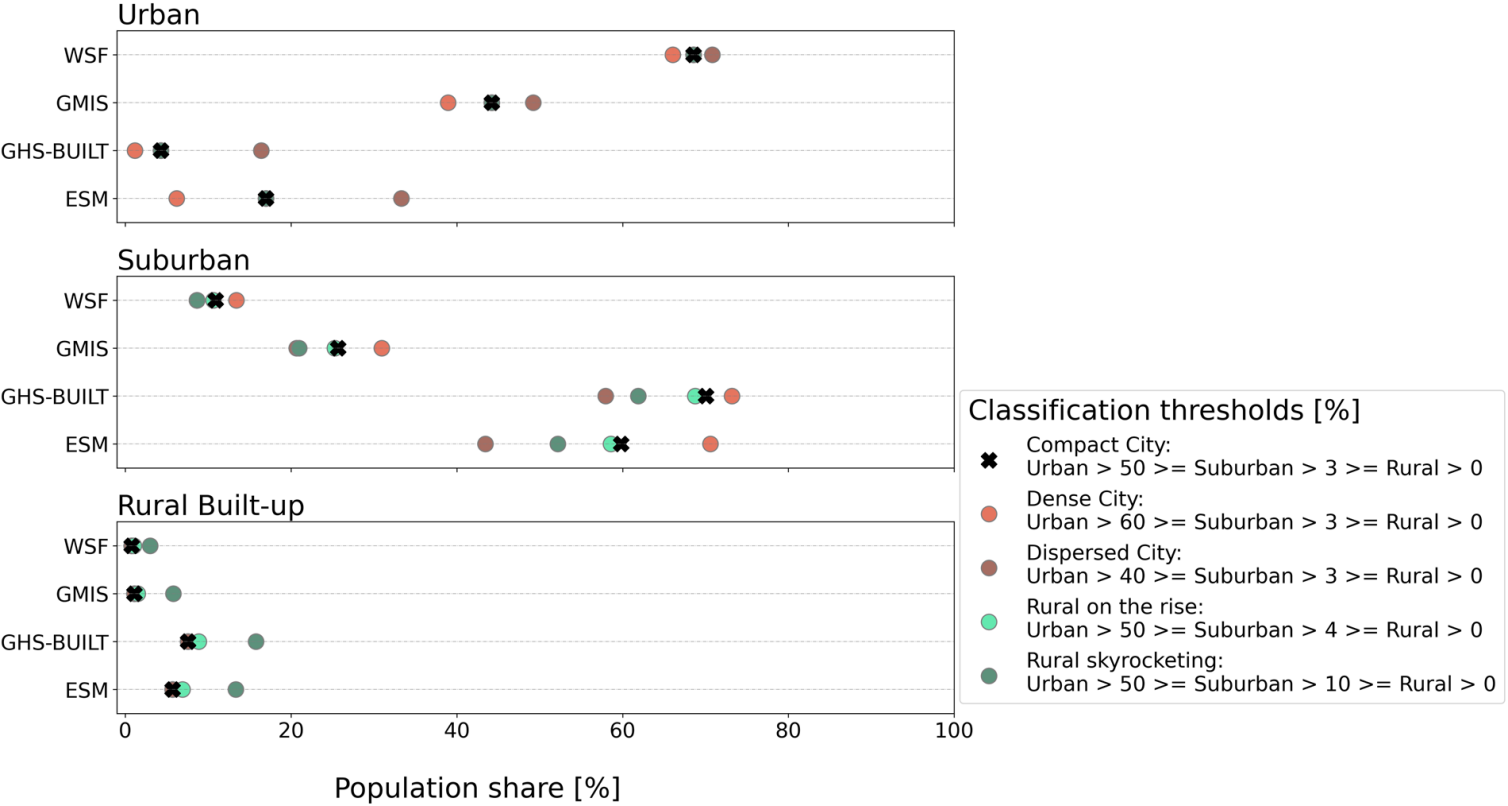
Changes in the estimates of exposed population per built-up area class for different threshold values. The different classification schemes are indicated by the colored circles. The classification scheme used within the study is represented as a black cross.

We tested the sensitivity of the different built-up proxy datasets to the settlement type thresholds by increasing or decreasing the upper and lower thresholds by up to 10% (see also Table 2 in the Methods section). Figure 4 shows that the different built-up proxy datasets show varying sensitivities towards changes in the settlement type thresholds. In the “Urban” class the population share is increasing/decreasing when the upper threshold is lower/higher. WSF is the least sensitive to threshold changes with a difference in exposed population of 5% between the classification schemes, whereas ESM has the highest difference with 27%, which corresponds to an absolute difference of 9 and 53.5 million people respectively. For the “Suburban” class larger shares of population are estimated when the range between upper and lower threshold increases. Therefore, the “Dense City” scheme yields the highest results and the “Dispersed City” scheme the lowest. Similar to the “Urban” class, WSF is the least and ESM the most sensitive to changes in the class thresholds. In the “Rural Built-up” class increasing the lower threshold leads to a higher population share, with the estimates for the “Rural skyrocketing” scheme being the highest and the “Compact City” scheme being the lowest population estimates. Also, for this class, WSF is the least sensitive to the threshold changes with a difference of 2% (4 million people), and GHS-BUILT the most sensitive with 8% (13.5 million people).

Overall, we observe the strongest impact of varying class thresholds on the population estimates in the “Urban” and “Suburban” class. Here, WSF is, in general, the least sensitive to the threshold adjustments, whereas the exposed population estimates vary the most for the ESM data.

## Discussion

### Different built-up area definitions can lead to differences in population exposure by up to 65%

Although exposed total built-up area and population count are very similar across all datasets, there are significant discrepancies in the population and area shares for the different settlement types. We see large differences in the population density per settlement type, with up to 65% difference in the “Urban” class and 59% in the “Suburban” class. These differences result from the various data collection techniques and built-up area definitions used to create the built-up proxy datasets^30^ (Table 1). For instance, GHS-BUILT and ESM are both based on the same machine learning algorithm used for extracting building features^49^, however the datasets rely on different remote sensing imagery that, for example, have different spatial resolutions^49,50^, potentially leading to deviations when determining built-up area. Further, many of the datasets use ancillary variables such as Open Street Map or nighttime light data to delineate built-up structures, which introduces new sources of uncertainty and endogeneity issues^43,51^. The GMIS dataset for example employs nighttime lights to identify built-up structures, which are likely to result in larger built-up footprints because of scattering lights^43,52^. In our study we specifically focus on the effects of different applied built-up area definitions on the exposure estimates. In Fig. 1 we see that the distribution of population and area estimates from GMIS and WSF largely differ from the estimates of GHS-BUILT and ESM because of the divergent underlying built-up area definition, as well as the difference in the total amount of built-up areas. GHS-BUILT and ESM only classify buildings as built-up area, while the WSF and GMIS also include all impervious structures. This leads to higher population and area shares in the “Urban” class for WSF and GMIS, as built-up areas have a higher density when surrounding infrastructure is included in the density calculation. On the other hand, GHS-BUILT and ESM distribute larger shares of population and built-up area in de the “Suburban” class, as they depict lower built-up area densities, per definition. In rural areas the effect of different built-up area definitions is less pronounced, leading to differences of up to 7% in the “Rural Built-Up” class. We assume that the built-up area definition is less impactful here as surrounding infrastructure is generally less dense in rural areas.

### Areas of high built-up density are better represented than sparsely built-up areas

We investigate the distribution of built-up area across the full range of built-up densities without distinguishing different settlement types with the help of a cumulative built-up analysis (Fig. 3). The curves in Fig. 3 show a higher agreement of the built-up proxy data with the regional data for built-up densities included in the “Urban” class, assuming a common built-up area definition. Therefore, we expect the exposure estimates for built-up land to be more reliable for the “Urban” class than for the “Suburban” and “Rural Built-Up” class and that the built-up area estimates may be even more reliable if the threshold for the “Urban” class is higher. This is in agreement with studies reporting difficulties in detecting settlement structures in rural areas or distinguishing them from bare soil^44–46,53^. This is further supported by the mismatch of spatial patterns of population and built-up area, which has been observed in previous studies^54,55^ and is here indicated by the large share of population living in “Rural No Built-up” areas (Fig. 1). Even though we observe a better representation of built-up area for all built-up proxy datasets in densely built-up areas, we still observe high absolute differences in the area and population estimates between the built-up proxy datasets, particularly in the “Urban” and “Suburban” class, where population densities are higher. This points to the strong impact of built-up area definitions on the distinction of settlement types. Therefore, the choice of data could have the biggest impact on the estimation of population distribution in the “Urban” class compared to the other classes after all.

### Exposure estimates are less sensitive to the classification scheme for built-up datasets that include all man-made structures

We find that compared to the other built-up proxy data, population estimates based on the WSF data are least sensitive to modifying the limits of the settlement types, due to the underlying definition of built-up area. WSF and GMIS generally have a higher share of high built-up density areas, and the cumulative areas seem to be more evenly distributed across the built-up density spectrum as a result of the applied built-up definition (Fig. 3). Therefore, changing the class threshold does not have such a significant impact for the WSF and GMIS data, as for datasets with a less homogenous distribution of built-up area densities like ESM and GHS-BUILT. Other studies saw comparable changes in the estimates of urban areas when adjusting the thresholds. Koomen *et al*.^56^ for example observed a reduction in the urban area extent by up to 0.25 million km² if the urban threshold was changed from 50% built-up density to 75% (15 arc seconds resolution); Balk *et al*.^54^ found that in 2010 the number of global urban population increases by 13% if the built-up density threshold is lowered from 50 to 25%. The sensitivity analysis underlines once more the impact of different built-up area definitions on the distinction of settlement types and therefore on the sensitivity of exposure assessments. It should be noted that due to the different amounts of total built-up area per dataset, the sensitivity of the population exposure due to the threshold changes can be further influenced.

### Built-up area datasets showing building footprints are more suitable for population analysis than datasets that include all man-made surfaces

**Fig. 5:**
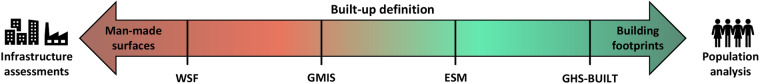
Ranking of the built-up proxy data suggesting suitable usage cases for the datasets.

Comparing the exposure estimates for each built-up area class with the estimates of the regional data (Figs. 2, 3), reveals that not all datasets may be used for all purposes, in light of the large differences in population and area estimates for each settlement type. The regional data chosen for the evaluation of our results apply different definitions of built-up land; therefore, alignment of the regional data with the built-up proxy data differ depending on the definition used. The regional data for Tallinn and Hamburg include man-made surfaces in addition to the building footprints, thus resulting in a higher agreement with the GMIS and WSF data. The regional data of Helsinki, Barcelona, Antwerp and Venice only depict building footprints and are therefore better aligned with GHS-BUILT and ESM. In our evaluation two sites (Hamburg, Tallinn), with a more extensive definition of built-up area, agree mostly with datasets including all impervious surfaces (WSF, GMIS); whereas the other four sites (Barcelona, Venice, Helsinki, Antwerp) are better aligned with GHS-BUILT and ESM as their definition of built-up area is alike. From these results we see that the definition of built-up areas largely influences the exposure estimates, which may be falsely interpreted if the built-up area definition is not regarded. Therefore, we suggest that for population analyses, calculating population exposure or projecting future population distributions, GHS-BUILT and ESM are more suitable. On the other hand, GMIS and WSF may be more suitable when looking into urban land use, such as the evaluation of human assets at risk or damage assessments of urban infrastructure. We frame our suggestion in a ranking, which we base on the size of population share per settlement type and the accordance of exposure estimates with the regional data, as well as the built-up proxy data’s sensitivity to the class thresholds (Fig. 5).

### Limitations in data alignment and biased regional data impact the analysis

The analysis is constrained by various limitations that need to be considered when interpreting the results. The results of our study are influenced by the choice of data. For example, for the population estimates we use the WorldPop unconstrained dataset, which distributes the population independent of the distribution of built-up area.^57,58^. While this avoids endogeneity issues when overlaying the population data with the built-up proxy data^43,51^; it results in a larger share of the population to be located in areas that are not considered as built-up area.

Further, the choice of the regional data introduces an additional bias to the analysis. The regional data are based on different built-up area definitions (Table 3), which are in better agreement with some of the built-up proxy datasets. Therefore, we do not recommend to use one specific built-up proxy dataset per se but to weigh the data choice based on the aim of the study, specifically considering the built-up area definition.

The combination of many different datasets leads to a spatial misalignment, especially at the coastline, which results in the misclassification of smaller population parts. We try to minimize errors introduced by the preprocessing of the data by employing data with the same spatial resolution and coordinate reference system (CRS). However, for example for the regional data, the preprocessing is inevitable because most data are provided in a vector format and have to be converted to a raster.

Additionally, the classification scheme of urban and rural classes needs to be considered when employing the data. Even though the classification thresholds we use for distinguishing different settlement types are based on previous literature, the sensitivity analysis shows that even small changes in the classification scheme can have large impacts on the exposure estimates.

### Final messages and outlook

Inaccurate estimates on exposure or estimates that are not put into context may lead to maladjusted local decisions^30^. Our study highlights the significant impact that the choice of built-up area data can have on population and built-up area exposure assessments, with differences in the estimates of population exposure of to up to 65% (“Urban” class). In addition, the distribution of exposed population within the settlement types can vary largely if the classification thresholds are modified. We argue that these differences in the results originate mostly from the different built-up area definitions of the datasets. Other data characteristics, such as the spatial resolution or the usage of ancillary variables to detect built-up land can further influence the distribution of exposure in different settlement types. Thus, our study emphasizes that datasets need to be chosen carefully and in a way that ensures that the collection methods and the utilization purpose of the data are compatible^21,30,41,42^. This is particularly relevant when integrating several datasets, as this increases the uncertainty of exposure assessments^54^. While we cannot recommend a single, most accurate dataset for all types of analysis, our comparison with regional data suggests that GHS-BUILT or ESM are preferable for population analysis, while GMIS and WSF are more suitable for comprehensive assessments of human infrastructure.

We want to stress the need to employ more refined urban classes in order to sufficiently capture settlement structures in between the dichotomous urban and rural classification. This is in line with the proposed indicators of several UN Sustainable Development Goals, which require a distinction into smaller urban units^32,40^. A more subdivided but consistent representation of the urban-rural continuum is important for distinguishing different settlement types for risk and damage assessments in the coastal zone, as the management of hazard impacts varies depending on different urban intensities^33,35^. The accelerated growth of suburban locations further underlines the urge for a refined definition of urban and rural land^56,59,60^. Future research should incorporate this extended urban distinction to facilitate more precise assessments of sparser urban areas. In this context it can be of additional interest to compare the settlement type distinction based on the built-up area density with the definitions of the DEGURBA or to investigate regional patterns of sensitivity towards the classification of different settlement types. In our analysis we also implemented the DEGURBA classification based on the WorldPop constrained data (Supplementary Figure 2). We observed that the DEGURBA classification distributes population more homogenously across the settlement types than the built-up proxy datasets. Still, as our analysis focuses on the effect of different built-up area data on the distribution of population exposure within different settlement types, independent of population data, we did not investigate this further. However, we have added some results in Supplementary Figure 2 that may trigger further research.

We find that the exposure estimates of population and built-up area for each built-up area class can vary largely depending on the built-up area dataset. However, built-up area is not the only factor influencing exposure analysis. As described by MacManus *et al*.^43^ the population data and DEM have an equally high contribution to the accuracy of the exposure estimates. Our study could be extended by assessing the impacts of additional data variables or analyze different datasets.

## Methods

Our study evaluates the exposure of population and built-up land within diverse settlement types in Europe’s coastal regions to climate hazards. The process involves defining different settlement types based on specific built-up area density thresholds and intersecting them with population and area data. We quantify the impact of the underlying built-up area data on the exposure estimates by applying the analysis to four different built-up area density datasets. We further compare the exposure estimates with regional built-up area data and conduct a sensitivity analysis by varying the density thresholds.

### Data selection

**Fig. 6:**
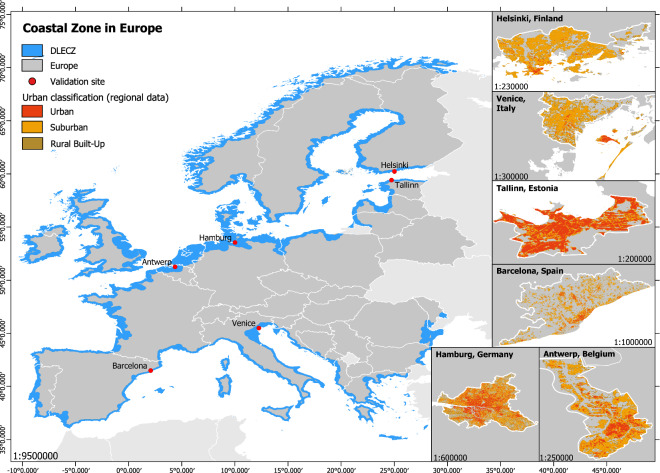
Map with all included European countries and the Distance Low Elevation Coastal Zone (DLECZ). On the right the cities used for the evaluation of the results are shown, classified into “Urban”, “Suburban” and “Rural Built-up” class according to the regional data.

In our study, we include all countries that geographically belong to continental Europe and for which population data are available. The analysis is therefore performed for 43 countries in total, of which 30 are adjacent to the coast (Fig. 6).

To estimate the physical and social exposure (i.e. population and built-up land) to climate hazards in distinct settlement types for different underlying built-up area datasets, we use gridded data on population, elevation and the built-up area density. Data properties are summarized in Table 1. Our selection criteria for population data are guided by two main considerations. First, we require a spatial resolution of at least 3 arc seconds to accurately capture the narrow coastal region, which we observe in most European countries. Second, the temporal resolution should align closely with the built-up proxy data, ideally around the year 2015. Based on these criteria, we exclude datasets such as HYDE (10 km resolution), Landscan (1 km resolution), GPW (1 km resolution) and Eurostat Census Grid (1 km resolution, available only for 2021 and EU-countries), leaving three potential options: WorldPop constrained and unconstrained, and GHS-POP. To determine the most suitable dataset, we conducted a sensitivity analysis (Supplementary Figure 3), assessing the European-wide exposure using each of the three population datasets. Our analysis showed that the population distribution across settlement types remains consistent regardless of the chosen population data. Therefore, we decided to utilize the WorldPop unconstrained dataset in 2015. Additionally, the WorldPop unconstrained data does not use built-up area data as a variable for redistributing the population census, like WordlPop constrained or GHS-POP^57,58^; therefore, the population estimates of the settlement types analyzed in this study are not influenced^43,51^. We define the coastal zone by expanding the definition of the commonly used Low Elevation Coastal Zone (LECZ), which describes the area that is below 10 m elevation and, which is hydrologically connected to the sea^1,2^. We extend the LECZ by a 20 km distance zone in order to include larger coastal cities^4^ and refer to the coastal zone as the Distance Low Elevation Coastal Zone (DLECZ)^39^. We employ the Multi-Error-Removed Improved Terrain (MERIT) Digital Elevation Model (DEM)^61^ to calculate the LECZ. From the inland boundary of the LECZ we generate a 20 km distance zone, which we combine with the LECZ to generate the DLECZ. For the built-up area data, we choose four different datasets, which are commonly employed in impact, vulnerability and adaptation studies and which have a spatial resolution, which is similar to the resolution of the population data (Table 1). Further it is important that the built-up area datasets depict built-up densities, in order to allow the delineation of different settlement types. These requirements are met by the Global Human Settlement – Built-up Surface grid (GHS-BUILT-S R2022A)^62^, the European Settlement Map (ESM)^63^, the World Settlement Footprint (WSF)^64^ and the Global Man-made Impervious Surface (GMIS)^65^ data (Table 1). All datasets provide the built-up area share per pixel, however each dataset is based on different satellite imagery and is available for different years, differing by up to five years. We did not account for this temporal shift in our analysis, however we assume that for European countries the change in built-up density within this period can be neglected. Furthermore, the GMIS data are provided at a higher resolution of 1 arc second. We minimized the effect of the spatial resolution mismatch by reducing the resolution to 3 arc sec after the classification of the settlement types. The main focus of our study is however on the evaluation of the different definitions and methods to delineate built-up areas: GHS-BUILT and ESM use building footprints to define built-up land^49,50^, whereas WSF and GMIS additionally include all man-made surfaces^45,66^. We first process the datasets in their original CRS and resolution and then align them to the WGS84 CRS and a resolution of 3 arc sec, which matches the specifications of the WorldPop data.

### Data sources

The characteristics of the datasets used in our analysis are summarized in Table 1. Here we provide the links for downloading the data.

Population data *WorldPop unconstrained* is available at 10.5258/SOTON/WP00645. The digital elevation model *MERIT DEM* is available at http://hydro.iis.u-tokyo.ac.jp/~yamadai/MERIT_DEM/. *CIESIN real area grid* is available at 10.7927/H48050JH. The built-up area proxy data:

*GHS-BUILT-S R2022A* is available at 10.2905/D07D81B4-7680-4D28-B896-583745C27085. *GMIS* is available at 10.7927/H4P55KKF. *ESM* 2016 is available at https://land.copernicus.eu/pan-european/GHSL/european-settlement-map/EU%20GHSL%202014?tab=download. *WSF 2015* is available at 10.6084/m9.figshare.c.4712852.v1.

The regional built-up area data:

*Historical Settlement Data Compilation for Spain* is available at 10.6084/m9.figshare.22009643. *Land cover fraction map of Germany* is available at 10.1594/PANGAEA.920894. *Buildings 3D Finland* are available at https://asiointi.maanmittauslaitos.fi/karttapaikka/tiedostopalvelu/3d-rakennukset. *3D-model of the City of Antwerp* is available at https://s3-ant1.antwerpen.be/prd-3d/index.html. The building footprints of Venice *Edifici Veneto* are available at https://idt2.regione.veneto.it/geoportal/catalog/search/resource/details.page?uuid=r_veneto:edifici_veneto. The building 3D model data 2017 of Estonia are available at https://geoportaal.maaamet.ee/eng/Download-3D-data-p837.html.

**Table 1:** Specifications of the datasets used to generate the coastal zone and calculate the population and area exposure estimates for different settlement types, which are defined by different built-up area data.

Variable	Dataset	Derived from (base data)	Specifications [Unit]	Method	Built-up definition	Resolution	Time Steps	CRS
**Population**	**WorldPop**^78^	Gridded Population of the World (GPW) census counts	Population [count] **Unconstrained**/constrained	Random forest-based dasymetric redistribution of census counts based on ancillary variables	—	30 arc sec (~1 km) **3 arc sec (~100 m)**	2000 – 2020, **2015**	WGS84
**Elevation**	**MERIT DEM**^61^ Multi-Error-Improved-Terrain DEM	SRTM3 v2.1 AW3D30 v1	Terrain elevation [m]	Removes error components from existing DEMs	—	**3 arc sec (~100** **m)**	2000 (SRTM) 2006–2011 (AW3D30)	WGS84 EGM96
**Area**	**GPWv3**^68^ Gridded Population of the World Version 3: Land Area Grids	—	Global land area [km²]	—	—	2.5 arc minutes	2000	WGS84
**Built-up proxy**	**GHS-BUILT**^62^ Global Human Settlement – Built-up version 2022	**Landsat** (2018: Sentinel 2)	built-up density [%] Residential or non-residential	Machine learning algorithm	building footprints	10 m, **100 m**, 1 km	1975–2030 (every 5years) + 2018	World Mollweide
	**GMIS**^65^ Global Man-made impervious Surface	Landsat	built-up density [%]	Machine learning algorithm	building footprints & impervious surfaces	**30 m**, 250 m, 1 km	**2010**	WGS84
	**ESM**^63^ European Settlement Map **1. Release 2016**; 2. Release 2017 3. Release 2019	**1 + 2. SPOT 5 + 6** 3. Copernicus Very High-Resolution Imagery (VHR)	**1. Built-up density [%]** 2. Built-up area [extent] 3. Residential and non-residential built-up	Machine learning algorithm	building footprints	1. 10 m, 100 m 2. 2,5 m, 10 m, **100 m** 3. 2 m, 10 m	1 + 2. **2012** 3. 2015	ETRS89/LAEA
	**WSF**^64^ World Settlement Footprint **1. WSF2015**; 2. WSF2019; 3. WSF Evolution	**1. and 3. Landsat-8 and Sentinel-1** 2. Sentinel-1 and -2	**1. Built-up density [%]** 2. + 3. Built-up area [extent]	Machine learning algorithm	building footprints & impervious surfaces	10 m 1. **100 m**, 250 m,500 m, 1 km, 10 km	1985 - **2015**	WGS84

Some datasets include several versions, which are numbered consecutively. The characteristics printed in bold represent the version of the dataset that was used for the study.

### Estimating exposure per settlement type

To assess the exposure to coastal hazards in different urban settings we define four distinct settlement types based on built-up area density and combine the classes with population and area data. The class thresholds that we use to distinguish the settlement types have been used in previous studies^54,56,67^ and our classification scheme specifically refers to the study of MacManus *et al*. (Table 2)^43^. Built-up densities higher than 50% are assigned to the “Urban” class, and between 3% to 50% densities are defined as the “Suburban” class. A density below or equal to 3% is assigned to the “Rural” class, which is further subdivided into “Rural Built-up” (between 3% to 0%) and “Rural No Built-up” (below 0%) (Table 2). We process the data for the extent of the DLECZ to assess the exposed built-up area and population at the coast. We then calculate the exposed population and built-up area per settlement type by overlaying the built-up area class raster with the WorldPop data and a real area grid (i.e. Land and Geographic Unit Area Grid)^68^. As the built-up proxy data and the population data do not share the same coastline we assign the population shares located in the ocean equally to the three settlement types (“Urban”, “Suburban”, “Rural Built-up”).

We decided to distinguish the settlement types based on built-up area density instead of employing the DEGURBA because the focus of our study is to investigate the impact of different built-up area datasets on the exposure estimates, and not the effect of different population data. Therefore, we opted for an approach independent of population data to distinguish different settlement types. The DEGURBA classification uses population data to delineate different settlement types which may lead to endogeneity issues within population analysis^51^.

### Regional evaluation and sensitivity analysis

We compare the built-up proxy datasets with built-up area data from regional studies and authorities (Table 3)^69–74^. We choose six different European cities to regionally evaluate our results, that cover all geographical regions in Europe, namely Antwerp (Western Europe), Barcelona and Venice (Southern Europe), Hamburg (Central Europe), Tallinn (Eastern Europe) and Helsinki (Northern Europe) (Fig. 1). Most regional data are available in vector format; therefore, we convert the data from vector to raster showing the built-up density per pixel. For this purpose, we rasterize the vector data to 1 m resolution and overlay it with a 100 × 100 m grid to calculate the built-up area share. Afterwards we project the raster to geographic coordinates (i.e. WGS84) and 3 arc sec resolution. For Barcelona and Hamburg raster data depicting the built-up area density already exist at a resolution of 100 m^75,76^. The spatial extent of the settlement types per regional data is visualized in Fig. 6. To compare the regional data with the built-up proxy data, we apply the same classification scheme (Table 2) to the regional data and calculate the area and population shares per built-up area class.

Besides the exposure distribution within each built-up area class we also investigate the distribution of the built-up area along the full built-up area density range. In this manner we can evaluate whether the choice of the thresholds can be optimized. Hereby, we use built-up density curves as introduced by Florczyk *et al*.^49^. The curves illustrate the relationship between the built-up area density and cumulative built-up area. To generate a cumulative built-up density curve, the area per density percentage point (1 to 100%) is calculated and aggregated from the highest to the lowest density. This means that from higher to lower built-up densities the area share increases continuously so that finally at 1% density the total built-up area is depicted by the curve. As we generate the built-up density curves for all built-up proxy datasets and the regional data we are able to observe for which built-up densities the built-up proxy data is most aligned with the regional data.

Last, we test the sensitivity of the population estimates by varying the class thresholds. In total, we estimate the population exposure for four divergent classification schemes, changing the upper and lower thresholds by up to 10% as, based on the literature, we consider this a reasonable range to adjust the thresholds. We name the classification schemes according to their effect on the settlement compactness (Table 2). The entire analysis described in the methods sections is only performed for the “Compact City” classification scheme.

**Table 2:** Built-up area density thresholds defining the settlement types for five different classification schemes.

Classification Scheme	Urban	Suburban	Rural
**Compact City**	**>50%**	**< = 50% and >3%**	**< = 3% and >0%**
Dense City	>60%	< = 60% and >3%	< = 3% and >0%
Dispersed City	>40%	< = 40% and >3%	< = 3% and >0%
Rural on the Rise	>50%	< = 50% and >4%	< = 4% and >0%
Rural skyrocketing	>50%	< = 50% and >10%	< = 10% and >0%

The “Compact City” classification scheme is the scheme used throughout the entire analysis. The other four schemes are used to test the sensitivity of the population estimates to density threshold changes.

**Table 3:** Specifications of the regional data.

Variable	Dataset	Location	Derived from (base data)	Specification	Built-up definition	Resolution	Time Steps	CRS
**Built-up validation**	**HISDAC-ES**^69^ Historical Settlement Data Collection	Spain	Cadastral data	Building density evolution	Building footprints	100 m	1900–2020 (2015)	ETRS89/LAEA
	**Schug** ***et al****.*^70^	Germany & Austria	Sentinel 2 spectral temporal metrics	Landcover fractions for built-up Surfaces and infrastructure, Woody vegetation and non-woody vegetation	Building footprints and infrastructure	10 m	2018	ETRS89/LAEA
	**Buildings 3D**^71^	Finland	Laser scans from the National Topographic Database (KMTK)	3D building data	Building footprints	Vector data	2022	ETRS89/TM35FIN
	**3D-model stad Antwerpen**^72^	Antwerp, Belgium	Regional DEM (Digitaal Hoogtemodel Vlaanderen 2014) & digitial topographic reference map (GRB)	3D building data	Building footprints	Vector data	2014	Belge 1972/Belgian Lambert 72
	**Edfifici del Veneto**^73^	Venice, Italy	Cadastral data (CTR - Carta tecnica regionale) and regional DTM	Building footprints	Building footprints	Vector data	2022	RDN2008 - Zone 12NE
	**LOD1**^74^ Building 3D models	Estonia	Lidar elevation data (ALS) and building footprints (Estonian Topgraphic Database)	3D building data (flat roof)	Building properties	Vector data	2017	Estonian Coordinate System of 1997

### Supplementary information

Supplementary Material

## Data Availability

Dataset specifications and references of the input data are provided within the paper. A layer depicting the DLECZ, as well as layer showing the settlement types based on the different built-up area datasets on a European and City scale are available at 10.6084/m9.figshare.c.6839949^77^.

## Code availability

The python code to create the DLECZ, classify the settlement types and create built-up area density maps from building vector data in order to validate the results, is available at 10.6084/m9.figshare.c.6839949^77^.
