# Supplementary material for: Comparing built-up area datasets to assess urban exposure to coastal hazards in Europe
Source: Sci Data. 2024 May 15;11:499. doi: 10.1038/s41597-024-03339-4 (PMC11096343; doi:10.1038/s41597-024-03339-4)
Supplement: Supplementary file 1 — Supplementary Material [file 41597_2024_3339_MOESM1_ESM.docx]

# Supplementary Material


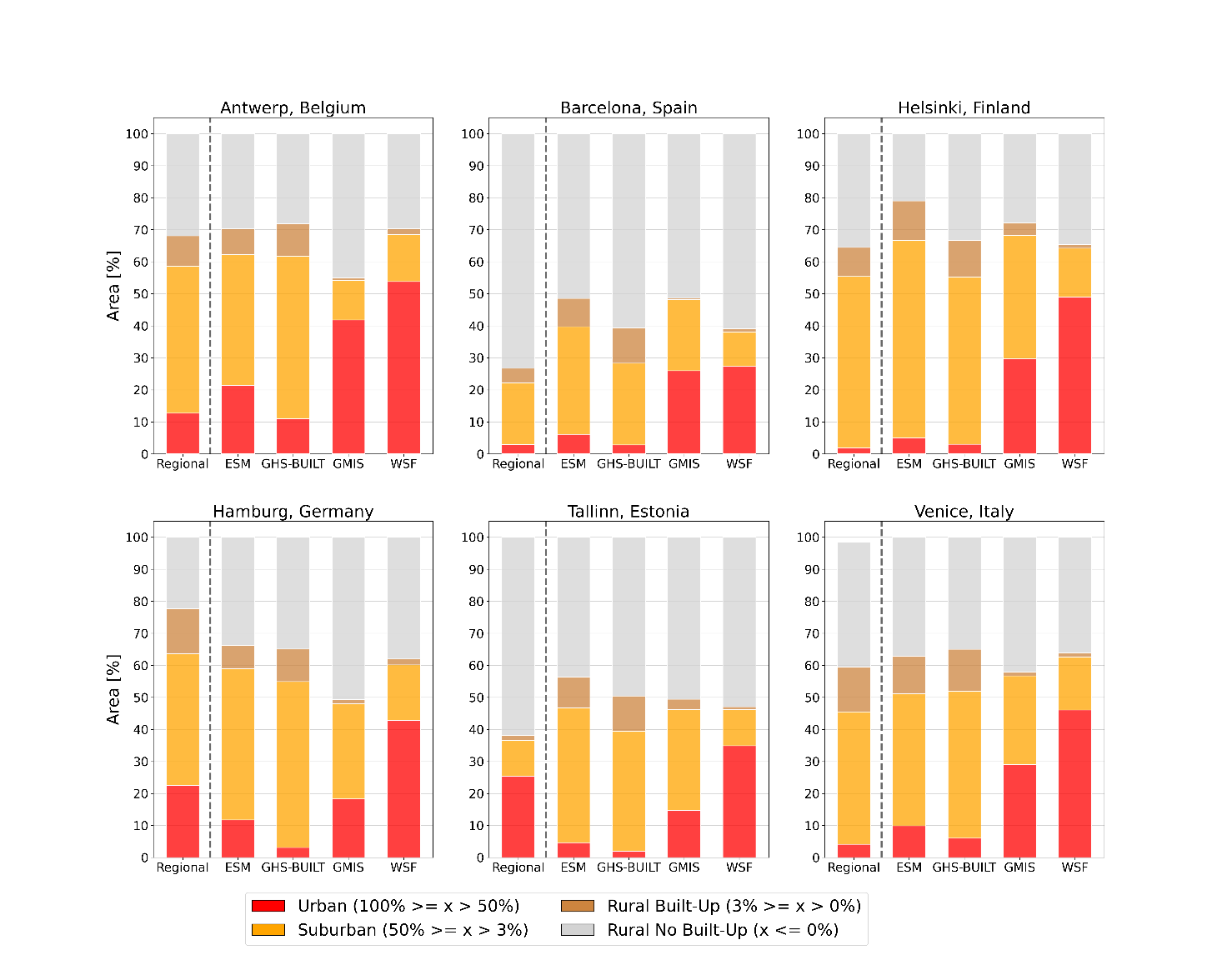


Supplementary Figure 1: Comparison of the relative built-up area shares per settlement type estimated by the built-up proxy data and the regional data for six European cities.


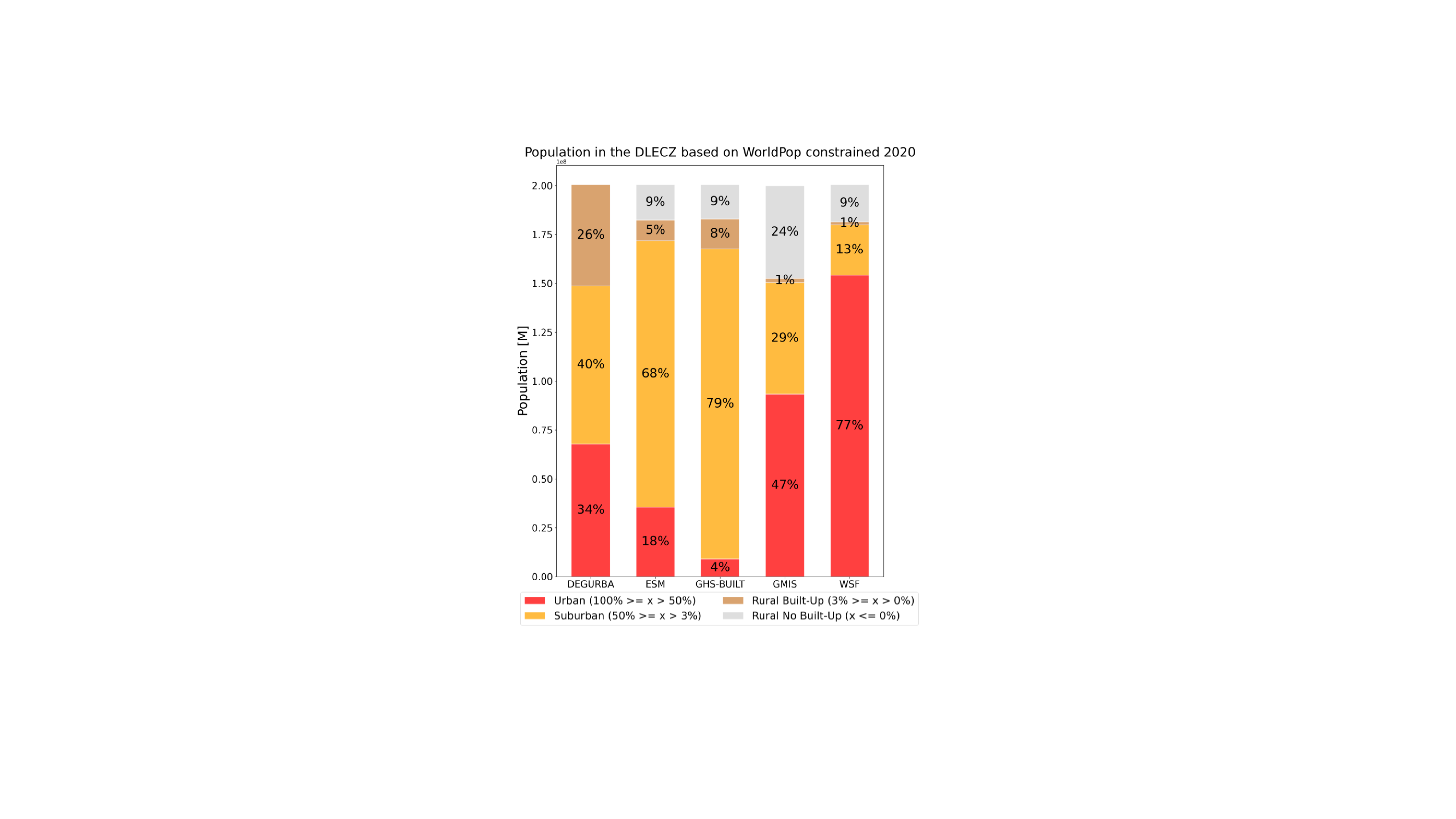


Supplementary Figure 2: Distribution of coastal population in different settlement types, which are defined based on the Degree of Urbanisation approach, using a population-based definition (left bar), or based on the built-up area density depicted by different built-up area datasets.


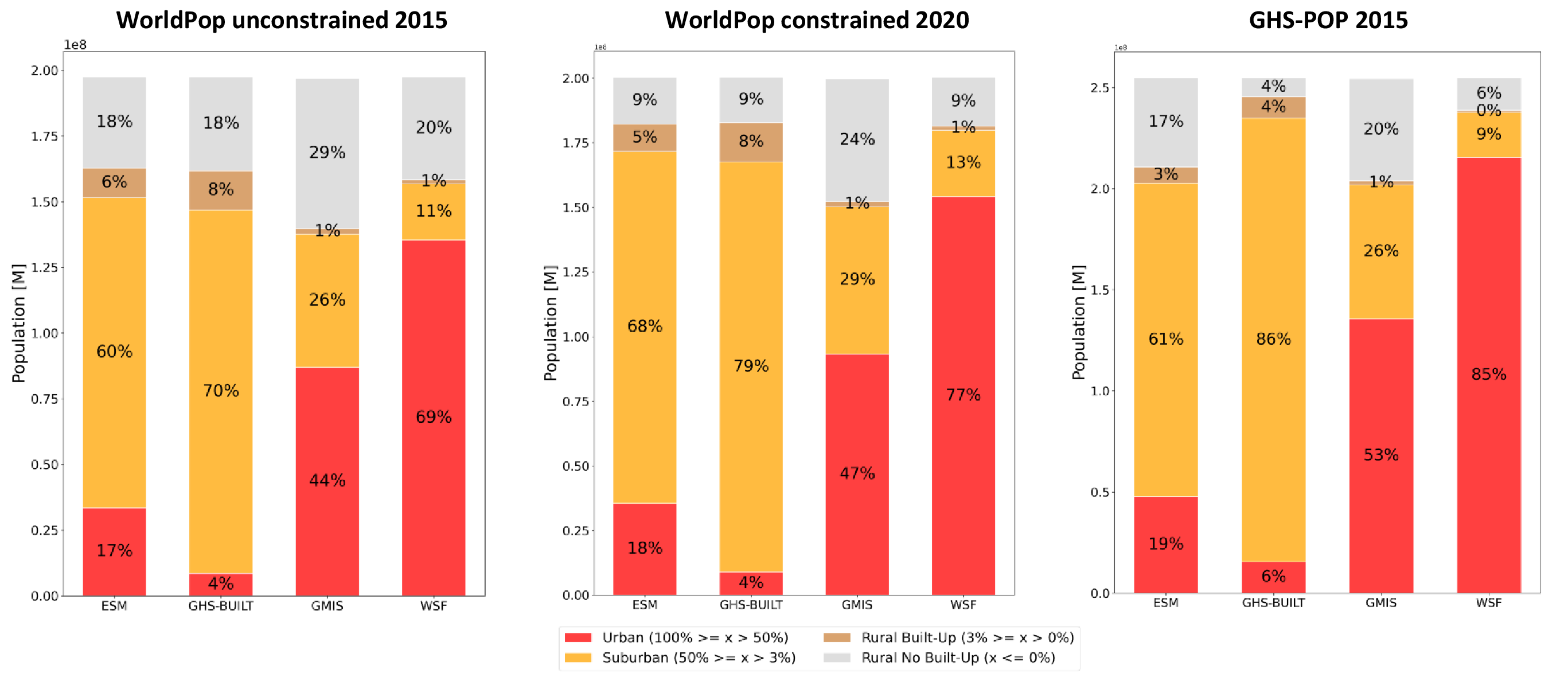


Supplementary Figure 3: Population exposure in the coastal zone of Europe for settlement types defined by the built-up area density of four different built-up area datasets. The left figure uses WorldPop unconstrained for the exposure assessment, the middle figure WorldPop constrained and the right GHS-POP.
